# Adenosine-A_2A_ Receptor Signaling Plays a Crucial Role in Sudden Unexpected Death in Epilepsy

**DOI:** 10.3389/fphar.2022.910535

**Published:** 2022-06-09

**Authors:** Hai-Ying Shen, Sadie B. Baer, Raey Gesese, John M. Cook, Landen Weltha, Shayla Q. Coffman, Jie Wu, Jiang-Fan Chen, Ming Gao, Teng Ji

**Affiliations:** ^1^ Department of Neuroscience, Legacy Research Institute, Portland, OR, United States; ^2^ Department of Neurobiology, Barrow Neurological Institute, St. Joseph’s Hospital and Medical Center, Phoenix, AZ, United States; ^3^ Molecular Neuropharmacology Laboratory, School of Optometry and Ophthalmology and Eye Hospital, Wenzhou Medical University, Wenzhou, China; ^4^ Department of Pediatric Neurology, Randall Children’s Hospital, Legacy Emanuel Medical Center, Portland, OR, United States

**Keywords:** adenosine A_2A_ receptor, NTS, nucleus tractus solitarius, brainstem, SUDEP (sudden unexplained death in epilepsy), local field potential, adenosine kinase

## Abstract

Adenosinergic activities are suggested to participate in SUDEP pathophysiology; this study aimed to evaluate the adenosine hypothesis of SUDEP and specifically the role of adenosine A_2A_ receptor (A_2A_R) in the development of a SUDEP mouse model with relevant clinical features. Using a combined paradigm of intrahippocampal and intraperitoneal administration of kainic acid (KA), we developed a boosted-KA model of SUDEP in genetically modified adenosine kinase (ADK) knockdown (Adk^+/-^) mice, which has reduced ADK in the brain. Seizure activity was monitored using video-EEG methods, and *in vivo* recording of local field potential (LFP) was used to evaluate neuronal activity within the nucleus tractus solitarius (NTS). Our boosted-KA model of SUDEP was characterized by a delayed, postictal sudden death in epileptic mice. We demonstrated a higher incidence of SUDEP in Adk^+/-^ mice (34.8%) vs. WTs (8.0%), and the ADK inhibitor, 5-Iodotubercidin, further increased SUDEP in Adk^+/-^ mice (46.7%). We revealed that the NTS level of ADK was significantly increased in epileptic WTs, but not in epileptic Adk^+/-^ mutants, while the A_2A_R level in NTS was increased in epileptic (WT and Adk^+/-^) mice vs. non-epileptic controls. The A_2A_R antagonist, SCH58261, significantly reduced SUDEP events in Adk^+/-^ mice. LFP data showed that SCH58261 partially restored KA injection-induced suppression of gamma oscillation in the NTS of epileptic WT mice, whereas SCH58261 increased theta and beta oscillations in Adk^+/-^ mutants after KA injection, albeit with no change in gamma oscillations. These LFP findings suggest that SCH58261 and KA induced changes in local neuronal activities in the NTS of epileptic mice. We revealed a crucial role for NTS A_2A_R in SUDEP pathophysiology suggesting A_2A_R as a potential therapeutic target for SUDEP risk prevention.

## Introduction

SUDEP is the leading cause of death in individuals with epilepsy, and as yet, no pharmacological intervention is available (Surges et al., 2009; Devinsky et al., 2016; Maguire et al., 2016). Although the mechanisms underlying SUDEP remain elusive, brainstem-related central apnea and cardiac arrest are considered two characteristic hallmarks (Stöllberger and Finsterer, 2004; Hirsch, 2010; Shorvon and Tomson, 2011). Animal models of SUDEP currently remain limited (Devinsky et al., 2016; Pansani et al., 2016; Li and Buchanan, 2019) in representing key features of clinical SUDEP cases, (i.e., the chronic nature of epilepsy and the preceding convulsive seizures), which in turn, impedes efforts in investigating SUDEP mechanisms.

The adenosinergic system has been proposed as one of the potential mechanisms for the pathophysiological development of SUDEP (Shen et al., 2010; Massey et al., 2014; Devinsky et al., 2016; Faingold et al., 2016; Kommajosyula et al., 2016; Ashraf et al., 2021). Findings from animal studies and clinical evidence also suggest a complexity of adenosinergic adaptations in epilepsy and SUDEP, including changes in adenosine metabolism and adenosine receptors (ARs). For instance, adenosine A_1_ and A_2A_ receptors (A_1_R and A_2A_R) are identified to express on synapses in limbic cortical areas (Tetzlaff et al., 1987; Rebola et al., 2003a; Rebola et al., 2005a; Rebola et al., 2005b); an increased A_2A_R density and a decreased A_1_R density are shown at excitatory terminals of different limbic areas from animal models of epilepsy and patients with epilepsy (Rebola et al., 2003b; Rebola et al., 2005c; He et al., 2020). The disrupted adenosinergic system, e.g., altered densities of A_1_R, A_2A_R, and the adenosine metabolic enzyme, adenosine kinase (ADK) were also seen in different brain areas in patients with temporal lobe epilepsy (TLE) and correlated to SUDEP risk (Patodia et al., 2020). Thus, further characterization of brain area-dependent changes is warranted to reveal the complexity of adenosine and A_2A_R-mediated regulation actions in SUDEP. During seizure events, adenosine is increased by tremendous consumption of ATP, and sequentially, increased adenosine alters neurotransmission in the brain and acts as a potent endogenous anticonvulsant to terminate seizures (During and Spencer, 1992; Dunwiddie and Masino, 2001). This anticonvulsive effect is mainly due to A_1_Rs-mediated inhibition of excitatory neurotransmissions (Dunwiddie and Masino, 2001). However, seizure-induced increases of extracellular adenosine can also broadly affect brain regions outside the primary seizure origins to exert a wide spectrum of actions through dominantly distributed A_1_Rs in the hippocampus, cerebral cortex, and cerebellum and enriched A_2A_Rs in the striatum, nucleus accumbens, and brainstem (Fredholm et al., 2001; Sebastião and Ribeiro, 2009). Indeed, adenosine actions in the brainstem contribute to the central regulation of cardiorespiratory functions (Barraco and Janusz, 1989; Phillis et al., 1997) that are proposed to play a crucial role in SUDEP events (Hirsch, 2010; Massey et al., 2014; Devinsky et al., 2016). Since NTS is a critical hub for cardiorespiratory regulation, manipulation of A_2A_Rs and A_1_Rs in the NTS affects cardiac and respiratory functions (Minic et al., 2015). Specifically, activation of A_2A_Rs can alter GABAergic neuron activity (Wilson et al., 2004; Minic et al., 2015) and overactivation of A_2A_R was associated with increased mortality in mice with febrile seizures (Fukuda et al., 2012). These findings indicate an important role of A_2A_Rs and the brainstem in SUDEP pathophysiology.

The metabolic clearance of extracellular adenosine is important for limiting the activation of adenosine receptors and seizure-related death. Clinical studies showed upregulated ADK densities in the brains of TLE patients (Aronica et al., 2011). We previously demonstrated that inhibition of ADK led to increased sudden deaths in mice with acute seizures (Shen et al., 2010), indicating that adenosine removal is essential for the brain to respond to the seizure-induced adenosine surge in the seizing brain to maintain balanced adenosinergic activities, i.e., as an endogenous anticonvulsant affecting seizure risk. Of note, seizure-induced increases of extracellular adenosine were reported in patients with intractable epilepsy (During and Spencer, 1992), and importantly, patients with uncontrolled or refractory seizures are associated with a high risk of SUDEP (Sperling, 2001; Devinsky et al., 2016).

Taken together, we hypothesized that in the brainstem chronic seizure-induced adenosine surges, in combination with abnormal metabolic adenosine removal, may cause fatal overactivation of A_2A_R and result in cardiorespiratory dysfunctions and increased risk of SUDEP. The present study aimed to evaluate whether targeting A_2A_R activity can reduce SUDEP risk. To accomplish this objective, we developed a new SUDEP mouse model with relevant clinical features, using a boosted-KA administration paradigm, and we investigated the vulnerability of SUDEP phenotype in mutant Adk^+/-^ mouse (Boison et al., 2002; Palchykova et al., 2010) that was characterized by a ∼50% decrease in ADK and thus impaired adenosine clearance.

## Materials and Methods

### Animals and Pharmacological Reagents

All animal procedures were conducted in accordance with protocols approved by the Institutional Animal Care and Use Committee of the Legacy Research Institute (LRI, No. 120–2018, 114–2020, and 120–2021) and Barrow Neurological Institute (BNI, No. 366) consistent with the principles outlined by the National Institutes of Health. Adk^+/-^ mutants (Boison et al., 2002; Palchykova et al., 2010) and their wild-type (WT) littermates were bred at the LRI (Portland, OR, United States). Adult male mice (bodyweight of 26–30 g) were used for the experiments, which were housed in a temperature- and humidity-controlled room with a 12-h light/dark cycle (lights on at 7:00 a.m.) throughout the experimental period. The reagents used in this study were commercially purchased: Kainic acid (KA, 0222, Tocris), 5-Iodotubercidin (5-ITU, 1745, Tocris), SCH58261 (S4568, Sigma).

### Boosted Kainic Acid Model of SUDEP

**FIGURE 1:**
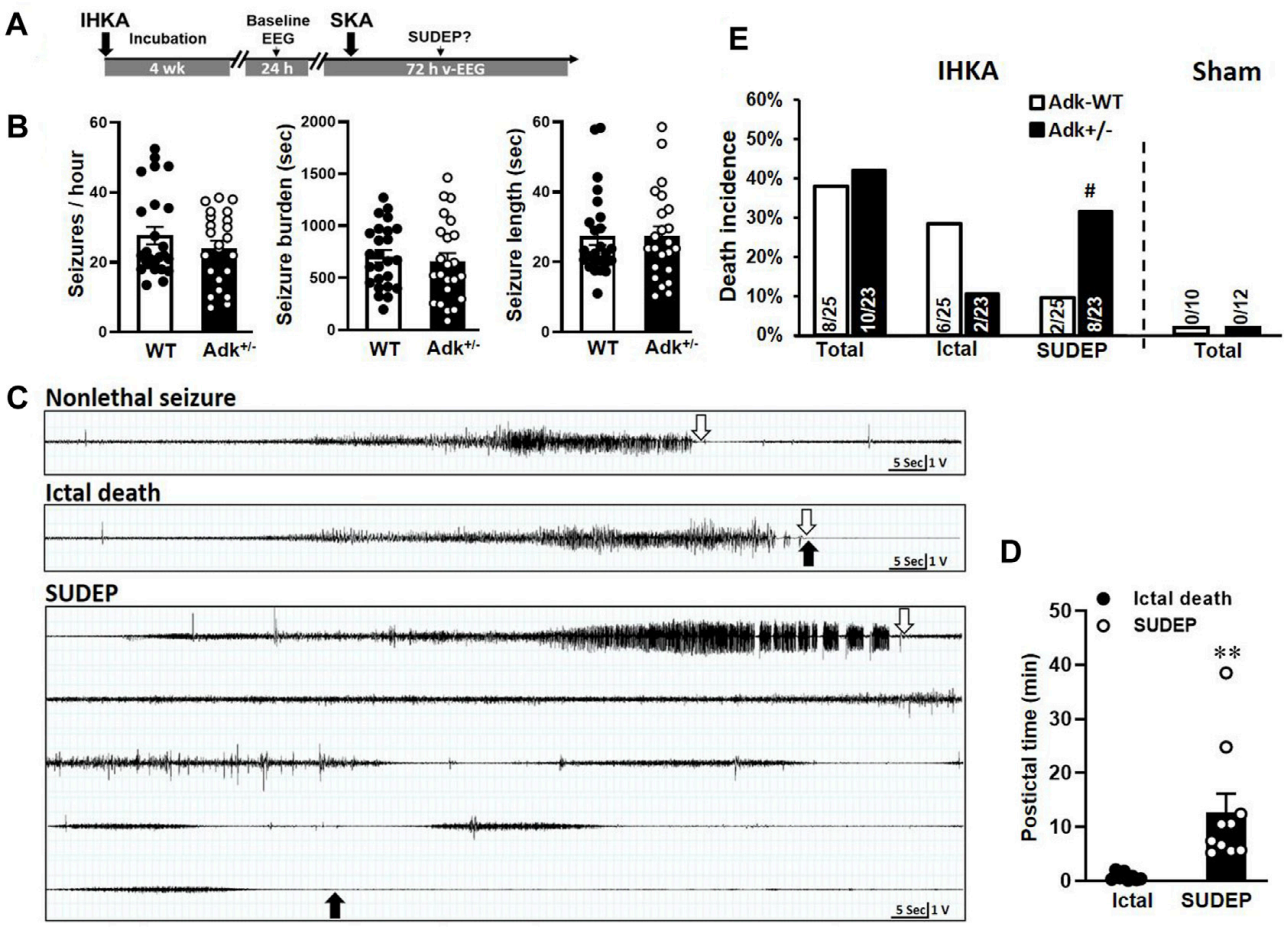
Boosted kainic acid (KA) model of SUDEP. **(A)** Boosted KA administration paradigm that consists of an intrahippocampal KA (IHKA) injection followed by a single systemic KA (SKA) intraperitoneal (i.p.) injection. **(B)** Baseline EEG evaluation of Adk^+/-^ mice and their WT littermates at 4 weeks after IHKA injection. **(C)** Representative EEG traces of non-lethal seizure (upper panel), ictal death (middle panel), and SUDEP event (lower panel). Open arrows indicate the stop points of seizures; solid arrows indicate the point of death occurrence. **(D)** The postictal period from the end of the seizure until the death occurred. **(E)** Rates of ictal death, SUDEP, and total mortality in mice received IHKA injection (IHKA) or vehicle control (Sham). Data are mean ± SEM. ^#^ *p* < 0.05 vs. SUDEP in Adk-WT, Fisher’s exact test, two-sided; **p < 0.01 vs. ictal death, unpaired *t*-test.

To develop a SUDEP model with clinically relevant features, we designed a boosted-KA administration paradigm that consists of an intrahippocampal KA (IHKA) injection followed by a single systemic KA (SKA) intraperitoneal (i.p.) injection (Figure 1A). Specifically, 1) for generating chronic epilepsy, adult male Adk^+/-^ and WT mice were subjected to unilateral IHKA (400 ng KA in 200 nl 0.9% saline—a dose used in our previous studies to establish chronic seizures (Shen et al., 2015)) or intrahippocampal injection of 200 nl saline (as sham control) into the right hemisphere, using coordinates (to Bregma): AP = −2.00 mm; ML = ± 1.25 mm; DV = −1.70 mm from procedures published previously (Shen et al., 2015). Ten days after the IHKA or vehicle injection, mice were implanted with bipolar coated stainless steel electrodes (80 μm in diameter; Plastics One) into the right hippocampus using the same coordinates as the IHKA injection (Shen et al., 2015). Animals were maintained in a group in their housing cage throughout the experiment period, except for the v-EEG monitoring period in which the mice were single-housed. 2) For triggering convulsive seizures and possible postictal SUDEP phenotype, one single SKA injection (15 mg/kg, i. p.—a dose that is not expectable to trigger convulsions in naïve mice) was given to the epileptic mice that received IHKA injection 6–8 weeks prior. Then, animals underwent a block of 72-h v-EEG recording for evaluation of possible SUDEP phenotype, and EEG seizure activities were also determined and analyzed (see next section).

### Video-Electroencephalogram Recording and Analysis

The video-electroencephalogram (v-EEG) was performed according to previously published methods (Shen et al., 2015). Mice were singly housed while tethered for the acquisition of the EEG recordings. Four weeks after IHKA injection, each mouse was subjected to a baseline EEG evaluation of an epileptic phenotype with a block of 24-h EEG monitoring and recording (P511/P122 Grass Instruments, Astro-Med, West Warwick, RI). Electrical brain activity was digitized (ML880 PowerLab 16/30; AD Instruments, Colorado Springs, CO) and quantification of EEG seizure activity was determined as in our previous work (Shen et al., 2015). For evaluation of possible SUDEP phenotype and pharmacological pretreatment on SUDEP risk, animals underwent a block of 72-h v-EEG recording, starting prior to the pretreatment of A_2A_R antagonist, SCH58261 (3 mg/kg), ADK inhibitor, 5-ITU (2 mg/kg), or vehicle (1.5% DMSO in saline) i. p. and lasting for 72 h. EEG seizure activities were determined and analyzed as aforementioned.

### Immunohistochemistry

For immunohistochemistry assessment of chronic epilepsy-induced biochemistry changes, a set of mice (n = 22) was sacrificed 6 weeks post-IHKA or vehicle injection after completion of EEG evaluation but without receiving systemic KA injection. Mice were transcardially perfused with 4% formaldehyde; the dissected brains were postfixed in 4% formaldehyde and cryoprotected in 30% sucrose PBS solution before sectioning into 30 μm sagittal sections using a cryostat (VT 1000 S, Leica, Bannockburn) (Shen et al., 2015). For staining, tissue sections were pretreated with citrate buffer at 80°C for 30 min; then blocked in goat blocking buffer (GBB, containing 2% goat serum, 0.05% Triton X-100 and 1% BSA) for 1 h and incubated at 1% sodium tetraborate for 30 min at room temperature. Pretreated sections were then incubated at 4°C for 48 h in GBB containing corresponding primary antibodies with the indicated dilution: ADK (A304-280A, Bethy Labs, 1:1,000), A_2A_R (A2A-GP-Af1000, Frontier Institute, 1:100), or cAMP (MAB2146, R&D Systems, 1:200); followed by incubation of corresponding secondary antibodies: Goat anti-guinea pig IgG H&L, Alexa Fluor 488 (A11073, Thermo, 1:500), Donkey anti-rabbit IgG H&L, Alexa Fluor 555 (A-31572, Thermo, 1:1,000), Donkey anti-mouse IgG H&L, Alexa Fluor 647 (ab150107, Abcam, 1:500), or NeuroTrace 435/455 Blue Fluorescent Nissl Stain (N21479, Thermo, 1: 1,000), for 90 min at room temperature. Sections were then washed and mounted on slides. Once dried, sections were cover-slipped with Vectashield Antifade Mounting Medium (H-1000) for fluorescence microscopy observation on a Leica TCS SPE confocal laser-scanning microscope (LAS X 3.1.2.16221). Three independent sections were stained for each method. All sections were processed in parallel using identical solutions and incubation times, while stain slices without either primary or secondary antibodies were used for controls.

### Image Quantification of Densitometry

**FIGURE 2:**
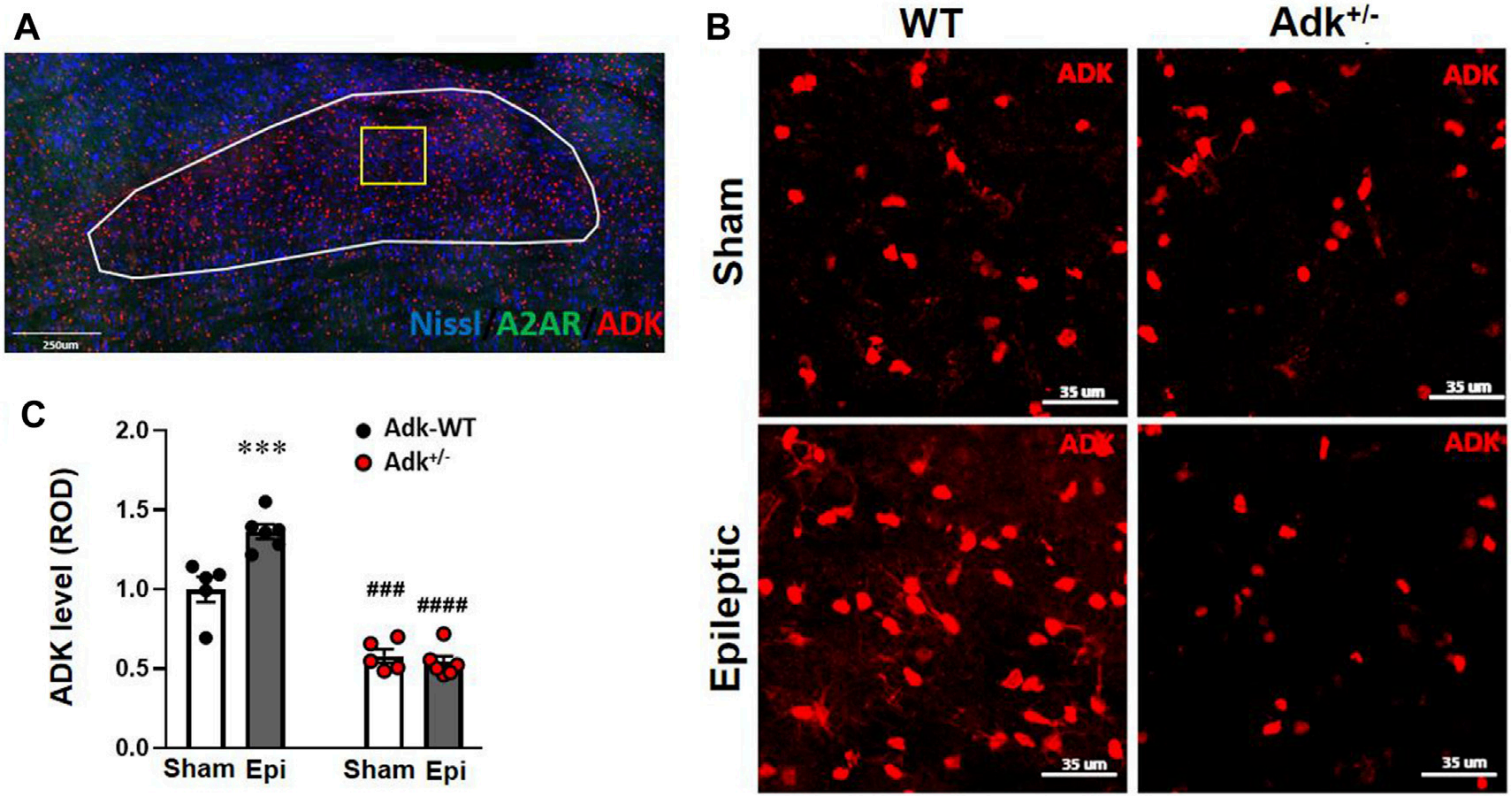
ADK changes in the NTS of IHKA modeled epileptic mice. **(A)** Representative immunofluorescence (IF) image and an indication of selection of NTS region. **(B)** Representative images of IF staining for ADK in the NTS of IHKA modeled epileptic mice (Epileptic) vs. sham controls (Sham). **(C)** Quantitative analysis of the NTS expression levels of ADK (presented as relative optical density, ROD) in IHKA modeled epileptic (Epi) mice vs. sham controls (Sham). Data are mean ± SEM. ***p < 0.001 vs. sham controls of same genotype; ^###^ *p* < 0.001 vs. WT sham controls; ^####^ *p* < 0.0001 vs. WT epileptics; unpaired *t*-test. Scale bar = 35 μm.

High-resolution digital images were acquired under identical conditions using the LasX software system (Leica, Buffalo Grove, IL, United States). Fluorescence intensity analysis was performed using Leica Application Suite analysis software (Leica, Buffalo Grove, IL) or ImageJ software (ImageJ, US. National Institutes of Health, Bethesda, MD; *ImageJ.nih.gov/ij/*). All image processing was applied identically across experimental groups. The NTS region was selected as shown in Figure 2A, and immunoreactive material was measured in the same designated area of NTS for each sample and expressed as relative optical density (ROD) by area. Three levels were measured for each mouse, and data analysis is expressed as the mean ± SEM of ROD. The average levels in treatment groups were normalized according to that in the corresponding control group (as baseline).

### Electrophysiology Recording of Local Field Potential in the NTS

*In vivo* LFP in the NTS was recorded with similar procedures as our previous work (Gao et al., 2007) using the below coordinates (to Bregma): AP = −7.35 mm; ML = 0.20 mm; DV = 4.50 mm, with animals under anesthesia (isoflurane; induction 3.0%, maintenance 1.5%). For LFP recordings, the signals were collected by tetrodes and amplified by a 16-channel amplifier (Plexon DigiAmp; bandpass filtered at 0.1–300 Hz, 2,000× gain, sampled at 2 k Hz). First, a baseline LFP was recorded for 30 min, then mice received pretreatment of SCH58261 (3 mg/kg, i. p.) or 0.9% saline (0.3 ml, i. p.) as control with continued recording; 30 min later mice were given a single KA dose (15 mg/kg, i. p.) and recording was continued for another 30 min. For LFP analysis, the raw data of the 2 min prior to the onset of drug administration were selected as representing ongoing LFP activity. A time-frequency transformation was performed (Hanning window; FFT size, 256) with NeuroExplorer, and the spectral power was calculated for each frequency resolution. The spectral power from all frequencies included within the bandwidth was averaged. LFP signals were divided into different frequency bands: theta (2–12 Hz), beta (15–35 Hz), and gamma (36–95 Hz).

### Statistical Analyses

All data were analyzed using GraphPad Prism software. The quantitative data are presented as mean ± SEM and were analyzed using one-way ANOVA, two-way ANOVA, or t-tests, as appropriate. The categorical data were analyzed using Fisher’s exact test or Chi-square test, as appropriate. A *p*-value < 0.05 was considered significant.

## Results

### Establishment of a Boosted KA Mouse Model of SUDEP

We established a new SUDEP model using a boosted-KA paradigm (Figure 1A) and tested the vulnerability of SUDEP risk in Adk^+/-^ mutants that have approximately 50% reduction in ADK protein level in the brain and compromised ability to metabolize adenosine (Boison et al., 2002; Palchykova et al., 2010). This model consists of two phases - chronic epilepsy modeled by IHKA injection (400 ng) and a potential phenotypic SUDEP (i.e., delayed postictal sudden death) event triggered by a single SKA (15 mg/kg) challenge (Figure 1A). We first assessed the epileptic features between Adk^+/-^ and WT mice at 4 weeks post-IHKA (or vehicle) injections as a baseline EEG evaluation. No EEG seizures were observed in sham control animals with the intrahippocampal injection of saline (WT n = 10 and Adk^+/-^ n = 12), whereas spontaneous recurrent electrographic seizures were developed in IHKA-injected WTs and Adk^+/-^ mutants. There was no significant difference in seizure-onset frequencies (*p* = 0.2778), seizure burden (i.e., total duration of seizure activity, *p* = 0.5995), and average length of seizures (*p* = 0.9687), between IHKA-injected Adk^+/-^ vs. WT mice (n = 23–25 per genotype, unpaired *t*-test, two-tailed) (Figure 1B).

After baseline EEG evaluations, mice were subjected to SKA (15 mg/kg, i. p.) to trigger possible SUDEP events. The v-EEG monitoring data showed SKA injection-induced convulsive seizures in all the epileptic animals, which eventually resulted in two outcomes: non-lethal seizures or lethal seizures (Figure 1C). Importantly, v-EEG monitoring demonstrated two distinctive phenotypes of lethal seizures: 1) ictal death - which occurred immediately after the end of SKA-induced seizures (Figure 1C, middle panel), or 2) delayed postictal death (aka, SUDEP event (Ryvlin et al., 2013)) - defined as a sudden death that occurred without coexisting behavioral and/or electrographic EEG seizures (based on v-EEG monitoring) for more than 5 min (Figure 1C, lower panel). In contrast, SKA (15 mg/kg, i. p.) did not trigger any death in non-epileptic sham control mice without prior IHKA (but intrahippocampal saline) injection, regardless of their genotypes (n = 10–12 per genotype) (Figure 1E, right panel). The v-EEG analysis showed that the SUDEP events occurred in a period of 12.73 ± 3.40 min after the last v-EEG-recorded seizure (Figure 1D), with the longest seizure-free period prior to a SUDEP event being 38.5 min. The periods from last seizure to death occurrence were significantly different between defined ictal deaths vs. SUDEP events (*p* = 0.0067, unpaired *t*-test, n = 8–10/phenotype) (Figure 1D). Remarkably, the occurrences of SUDEP events in epileptic Adk^+/-^ mutants (34.8%, 8/23) was significantly higher than epileptic WTs (8.0%, 2/25, *p* = 0.0335, Fisher’s exact test, two-sided), whereas the ictal seizure death rate was not significantly different between Adk^+/-^ mutants (8.7%, 2/23) and WTs (24.0%, 6/25) (*p* = 0.2487, Fisher’s exact test, two-sided), and the seizure-related total mortality was not significantly different between Adk^+/-^ mutants (43.5%, 10/23) and WTs (32.0%, 8/25) (*p* = 0.5524, Fisher’s exact test, two-sided) (Figure 1E, left panel). This suggests that Adk^+/-^ mice, with impaired adenosine removal, are more vulnerable to SUDEP risk.

### Chronic Epilepsy-Induced Adenosinergic Changes in the Brainstem of Mice

**FIGURE 3:**
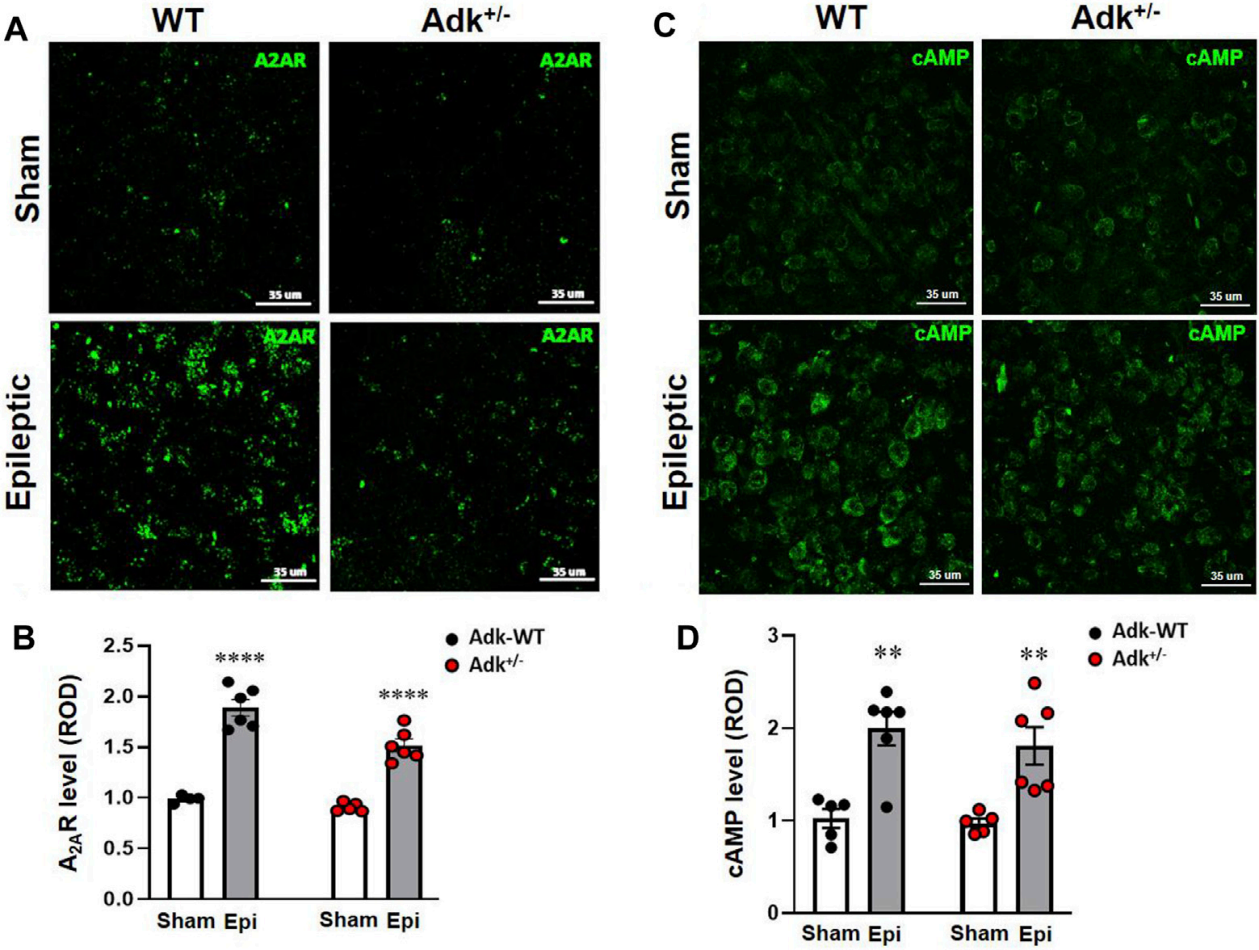
Changes of A_2A_R and cAMP in the NTS of IHKA modeled epileptic mice. Representative images of IF staining for A_2A_R **(A)** and cAMP **(C)** in the NTS of IHKA modeled epileptic mice (Epileptic) vs. sham controls (Sham). Quantitative analysis of the NTS densities of A_2A_R **(B)** and cAMP **(D)** (presented as relative optical density, ROD) in IHKA modeled epileptic (Epi) mice vs. sham controls (Sham). Data are mean ± SEM. **p < 0.01 and ****p < 0.0001, vs. sham controls within same genotype, unpaired *t*-test. Scale bar = 35 μm.

To understand the underlying mechanisms of chronic epilepsy-associated SUDEP, we evaluated the molecular changes related to adenosinergic activity with immunofluorescence staining of ADK, A_2A_R, and cAMP in the NTS. The NTS level of ADK (Figures 2B,C) was altered by *Adk* mutation and IHKA-modeled epilepsy (genotype factor, *p* < 0.0001 F_(1,18)_ = 189.2; modeling factor, *p* = 0.0012, F_(1,18)_ = 14.69; interaction, *p* = 0.002, F_(1,18)_ = 22.21; two-way ANOVA, n = 5–6 per group). Specifically, the basal NTS level of ADK in sham Adk^+/-^ mutants was lower than sham WTs (*p* < 0.0002), at 57.9% of the level of sham WTs (Figures 2B,C). The epileptic WTs had significantly (36.5%) increased NTS ADK vs. sham WTs (*p* = 0.0006, unpaired *t*-test). Of note, epileptic Adk^+/-^ mutants showed no increase in NTS ADK level vs. sham mutants (*p* = 0.9514, unpaired *t*-test), whereas NTS ADK level in epileptic Adk^+/-^ mutants was significantly lower vs. epileptic WTs (*p* < 0.0001, unpaired *t*-test) (Figures 2B,C). This suggests an epilepsy-induced compensatory increase of ADK in the NTS of WTs whereas Adk+/-mice were devoid of this change. Furthermore, NTS A_2A_R level was altered by IHKA epilepsy vs. sham controls (*p* < 0.0001, F_(1,18)_ = 166.5, modeling factor, two-way ANOVA, n = 5-6 per group), in both epileptic Adk^+/-^ and WT mice vs. their corresponding sham controls (*p* < 0.0001 and *p* < 0.0001, unpaired *t*-test) (Figures 3A,B **)**. These indicate an epilepsy-induced adaptive A_2A_R increase in the NTS of both genotypes, regardless of ADK levels.

To further explore A_2A_R activation-related changes, we evaluated cAMP in the NTS (Figure 3C). IHKA-modeled epilepsy significantly increased NTS cAMP levels (*p* < 0.0001, F_(1,18)_ = 32.17, treatment effect, two-way ANOVA, n = 5-6 per group) without a difference between genotypes (*p* = 0.4676, F_(1,18)_ = 0.5506, two-way ANOVA) (Figure 3D). Specifically, NTS cAMP levels were increased in both epileptic WTs and Adk^+/-^ mutant vs. their corresponding non-epileptic sham controls (*p* = 0.0022, *p* = 0.0080, *t*-test, n = 5–6 per group) (Figure 3D), which was in line with the changing pattern of A_2A_R in the NTS (Figure 3B). Together, these biochemistry findings revealed that chronic epilepsy resulted in increased ADK levels for the removal of excessive adenosine, and increased A_2A_R activities were in line with increased cAMP in the NTS.

### Blockade of A_2A_R Activation Reduced the SUDEP Risk in Epileptic Mice

**FIGURE 4:**
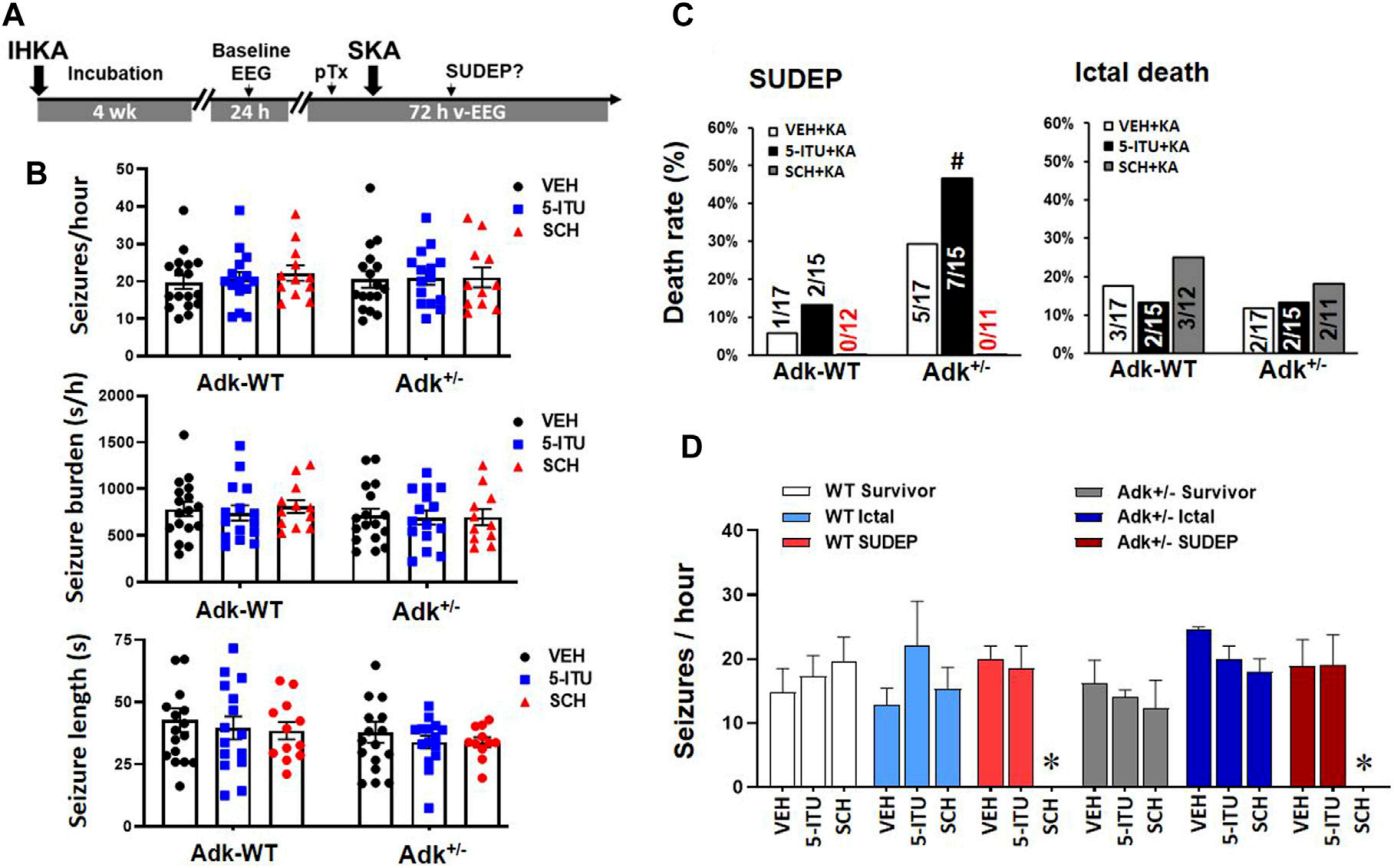
A_2A_R antagonist SCH58261 reduced SUDEP occurrence in epileptic mice. **(A)** The paradigm of pharmacological pretreatment (pTx) in the boosted KA model of SUDEP. **(B)** EEG validation of epileptic phenotypes in each group of Adk^+/-^ mice and their littermates (Adk-WT) at 4 weeks after IHKA injection, prior to the experimental SKA (15 mg/kg, i. p.) injections. **(C)** Rates of ictal death, SUDEP, and total mortality among groups with pretreatment of i. p. injection of vehicle (VEH), 5-Iodotubercidin (5-ITU, 2 mg/kg), and SCH58261 (SCH, 3 mg/kg). **(D)** Baseline EEG evaluation of seizure onset frequencies (seizure/hour) in mice grouped as survivors, ictal death, and SUDEP, with different pretreatments of a VEH, 5-ITU, or SCH. * Indicating zero animals with SUDEP existing in the marked (SCH) group. #*p* < 0.05 vs. same treatment group in Adk-WT mice, Chi-square test.

Having demonstrated perturbations in the adenosinergic pathway in the NTS induced by epilepsy, we further explored whether the increased A_2A_R activity contributes to the risk of SUDEP events. We tested if suppressing A_2A_R activity, with A_2A_R antagonist SCH58261, can reduce the risk of SUDEP and whether ADK inhibition can exacerbate SUDEP risk. A cohort of epileptic Adk^+/-^ mutants and WTs was generated by IHKA-injection and confirmed via EEG evaluation for their baseline epileptic features (Figure 4A). They then were randomly assigned to three groups to receive pretreatment of SCH58261 (3 mg/kg, i. p, n = 11–12), 5-ITU (2 mg/kg, i. p, n = 15), or vehicle (n = 17); and 30 min later each mouse was given a single injection of SKA (15 mg/kg, i. p.) followed by v-EEG monitoring for 72 h (Figure 4A). Figure 4B demonstrates the epileptic features of each group of mice at 4 weeks post-IHKA injections as their baseline EEG evaluation. There was no significant difference in seizure-onset frequencies between groups (n = 11–17 per group) (*p* = 0.7769, F_(2, 81)_ = 0.2532, two-way ANOVA) or between genotypes (*p* = 0.9867 F_(1, 81)_ = 0.0002, genotype effect, two-way ANOVA) (Figure 4B, upper panel); no significant difference was observed in seizure burden between groups (*p* = 0.8933, F_(2, 81)_ = 0.1130, two-way ANOVA) or between genotypes (*p* = 0.2321, F_(1, 81)_ = 1.450, genotype effect, two-way ANOVA) (Figure 4B, middle panel); also no difference was shown in average seizure length across groups (*p* = 0.5270, F_(2, 81)_ = 0.6456) and genotypes (*p* = 0.1338, F_(1, 81)_ = 2.294) (Figure 4B, lower panel).

The v-EEG analysis showed that the SUDEP occurrences increased in 5-ITU pretreatment (46.7%, 7/15), whereas SCH58261-pretreatment drastically reduced SUDEP events (0%, 0/11) in Adk+/-mutants vs. vehicle-pretreated controls (29.4%, 5/17) (X^2^_(2, N=43)_ = 6.901, *p* = 0.0317). The SCH58261-pretreatment also reduced SUDEP events in WTs (0%, 0/11), but 5-ITU pretreatment did not significantly increase SUDEP onset vs. vehicle pretreated WTs (X^2^_(2, N=44)_ = 1.904, *p* = 0.3860) (Figure 4C). These findings indicate that A_2A_R overactivation could contribute to increased SUDEP risk, and A_2A_R blockade efficiently reduced the SUDEP risk. Retrospective analysis of their baseline EEG showed that no significant differences were found in seizure onset frequencies among mice of survival, ictal death, and SUDEP groups that received different treatments (F_(5,10)_ = 1.799, *p* = 0.2007, one-way ANOVA) (Figure 4D). This suggests that the baseline epileptic phenotype was not linked to their seizure-related death phenotypes.

### A_2A_R Blockade Disinhibits Seizure-suppressed Coordination of Activity in the NTS

**FIGURE 5:**
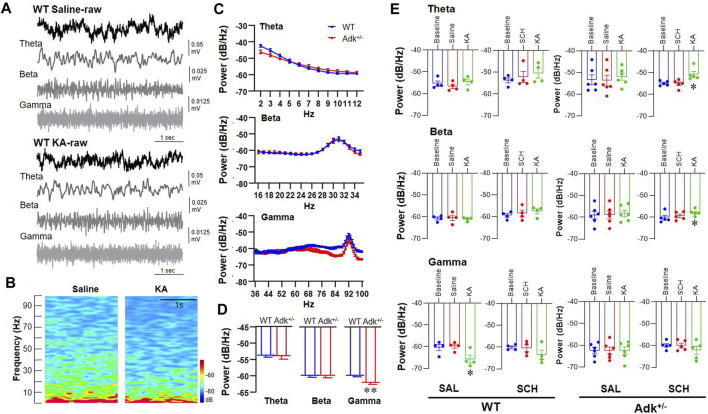
Electrophysiological LFP recording in the NTS of Adk^+/-^ and WT epileptic mice. **(A)** Typical LFP signals from one WT mouse after saline treatment (top) and subsequent KA injection (bottom). The first row of both upper (WT Saline-raw) and lower (WT KA-raw) panels showing a 5 s of the raw trace; the second to fourth rows in both panels showing a 5 s of filtered signals (Theta, Beta, and Gamma, respectively). **(B)** The power spectrum of the above LFP signals showing a reduced gamma response after KA injection. **(C)** The averaged power spectrums of LFP from WT and Adk^+/-^ mice, indicating a lower baseline gamma response in Adk^+/-^ mice. **(D)** The histogram showing a significant decrease in baseline gamma response in Adk^+/-^ mice. **(E)** Histograms of the averaged power spectrum of LFP signals showing quantitative changes in theta (top panel), beta (middle panel) and gamma (bottom panel) bands from WT and Adk^+/-^ mice after injections of saline followed KA or SCH followed KA. Data are mean ± SEM. *p < 0.05 vs. corresponding baseline, one-way ANOVA, in **(E)**; **p < 0.01 vs. WT baseline, paired *t*-test, in **(D)**.

To mechanistically dissect the A_2A_R effects on the coordination of activity in the NTS, SCH58261 or saline (as control) was administrated 30 min prior to KA injection in a separated cohort of IHKA-modeled Adk^+/-^ and WT mice (n = 4-6 per group), and LFP of NTS was recorded to reflect the neural activity of the assembly of cells surrounding the recording site (Bantikyan et al., 2009). We compared the LFP signals in epileptic Adk^+/-^ and WT mice following pretreatment with saline or SCH58261, and afterward with KA injections (Figure 5). The raw LFP signals were divided into three frequency bands, i.e., theta, 2–12 Hz; beta, 15–35 Hz; gamma, 36–95 Hz (Figure 5A). The heat map of the power spectrum of LFP signals shows an overt continuous reduced gamma response after KA in WT mice with saline pretreatment (Figure 5B). Analysis of averaged power spectrums between the genotypes demonstrated that the baseline oscillation power of gamma was significantly lower in Adk^+/-^ mice vs. WTs (*p* = 0.0037, unpaired *t*-test) while their baseline theta or beta powers were not different (*p* = 0.8656 and *p* = 0.9081, unpaired *t*-test, n = 5-6 per group) (Figure 5C).

Further analysis showed that the saline- or SCH58261-pretreatment *per se* did not cause LFP power changes in theta (F_(2,9)_ = 1.801, *p* = 0.2189), beta (F_(2,9)_ = 1.017, *p* = 0.3997), or gamma (F_(2, 9)_ = 0.0742, *p* = 0.9290, one-way ANOVA) band vs. baselines (Figure 5E). KA injection following saline pretreatment decreased the gamma oscillation in WTs (F_(2, 6)_ = 9.504, *p* = 0.0138, one-way ANOVA; *p* = 0.0077 vs. saline, *p* = 0.0115 vs. baseline, paired *t*-test), but the reduction of gamma oscillation was not seen when following SCH58261 pretreatment (F_(2, 6)_ = 3.057, *p* = 0.1215, one-way ANOVA; *p* = 0.0967 vs. SCH58261, *p* = 0.0328 vs. baseline, paired *t*-test), suggesting that SCH58231 partially blocked the KA suppression on gamma. The inferred role of A_2A_R is similar to that previously reported (Pietersen et al., 2009), although in this previous study kainate enhanced cortical gamma power. Also, while the baseline power of gamma oscillation in Adk^+/-^ mutants was significantly lower than WTs (Figure 5D), no gamma power change resulted after KA injection following saline pretreatment (F_(2, 10)_ = 0.0021, *p* = 0.9979, one-way ANOVA; *p* = 0.9509 vs. saline, *p* = 0.9649 vs. baseline, paired *t*-test) (Figure 5E, lower panel). Similarly, no KA-induced changes in theta (F_(2, 10)_ = 0.6913, *p* = 0.5234) or beta (F_(2, 10)_ = 0.3417, *p* = 0.7185, one-way ANOVA) were found in Adk^+/-^ mice when following saline pretreatment. Remarkably, the SCH58261 pretreatment significantly increased the theta (F_(2, 8)_ = 8.959, *p* = 0.0091, one-way ANOVA; *p* = 0.0056, paired *t*-test) and beta powers (F_(2, 8)_ = 5.452, *p* = 0.0321, one-way ANOVA; *p* = 0.0119, paired *t*-test) in Adk^+/-^ mice post-KA injection (Figure 5E, upper and middle panels). Together, we demonstrated the differential effects of KA-induced LFP power changes and SCH58261 manipulation on KA effects between WT and Adk^+/-^ mice.

## Discussion

While the mechanisms of SUDEP remain elusive (Stöllberger and Finsterer, 2004; Hirsch, 2010; Shorvon and Tomson, 2011), studies suggested that abnormalities in the adenosinergic system may play an important role in SUDEP events (Shen et al., 2010; Boison, 2012; Devinsky et al., 2016). We hypothesized that repeated seizure-induced adenosine increases in the brainstem can result in potentially fatal overactivation of A_2A_R while decreased ADK can exacerbate fatality. We developed a new SUDEP model characterized by a delayed postictal death phenotype in mice with chronic epilepsy. Our findings suggest an enhanced A_2A_R activity in the NTS of epileptic mice - while LFP alteration can result from a local modulation by A_2A_R and/or potentially by a long-distance network effect triggered by A_2A_R in different brain regions - and provide experimental evidence supporting A_2A_R as a therapeutic target for SUDEP prevention. To better understand the role of A_2A_R in SUDEP and its therapeutic potential, the following aspects warrant further discussion.

### SUDEP Animal Models and Clinically Relevant Phenotypes

The unmet need in developing pharmacological preventative therapies against SUDEP was not only due to inadequate understanding of SUDEP mechanisms (Hirsch, 2010; Shorvon and Tomson, 2011; Stöllberger and Finsterer, 2004) but also was compromised by limitations of available SUDEP animal models (Surges et al., 2009; Devinsky et al., 2016; Pansani et al., 2016). Of the previous animal models used for SUDEP research, most carry genetic mutations that mimic several major clinical conditions related to sudden death (e.g., cardiac arrhythmia, arrest, and coincidence of seizures) (Massey et al., 2014; Scheffer and Nabbout, 2019; Tu et al., 2011). Also, there is a lack of data in systematically characterizing the types of death (such as ictal death vs. delayed postictal death). These include rodents with mutations in the *SCN1A gene* (mimics Dravet syndrome) (Scheffer and Nabbout, 2019), *SCN5A gene* (mimics Brugada syndrome)*, KCNH2, KCNQ1 genes* (mimics Long QT syndrome), etc. (Tu et al., 2011). These genetic mutations could be potential causes of SUDEP; however, while they can result in pathophysiological changes, e.g., prolonged cardiac action potential, ventricular tachycardia, syncope, and sudden death, these genetic deficits were rarely reported in clinical SUDEP cases or patients with TLE—the most common form of epilepsy involved in SUDEP cases (Massey et al., 2014; Patodia et al., 2020; Devinsky, 2011). Facing these limitations, we developed a new SUDEP mouse model using a boosted-KA paradigm that mimics major, clinically relevant features of SUDEP cases, including: 1) a chronic nature of spontaneous seizures (Figure 1B) (Shen et al., 2015); and 2) a delayed postictal death phenotype - i.e., SUDEP event - one of the central revelations of the MORTEMUS study (Ryvlin et al., 2013), dissociated from prior convulsive and/or electrographic seizures (Figure 1C). Notably, the average latency to the SUDEP event is substantially longer than the delay seen in most (but not all) epilepsy patients reported in the MORTEMUS study (Ryvlin et al., 2013). A limitation of this study is the need for cardiopulmonary monitoring, which should be undertaken in future studies to fully characterize this model. It may be of interest to note that the adenosine modulation system also has direct cardiorespiratory effects which are affected in the global genetic and pharmacological manipulation attempted in this study. Additionally, future work could optimize our paradigm. For example, we utilized 15 mg/kg KA to trigger behavior seizures in epileptic mice; a lower KA dose may achieve a less severe behavioral seizure phenotype, while still triggering a SUDEP event in epileptic animals, resulting in a longer postictal period before SUDEP occurrence. These models have the potential for better characterization of adenosinergic changes in other brain regions in SUDEP, e.g., hippocampus, cortex, and other brainstem nuclei. Nevertheless, this adenosinergic SUDEP mouse model provides a novel tool for the SUDEP research field while it warrants continued optimization. The biological sex variable should also be investigated and addressed in future studies.

### Disturbances in the Adenosinergic Signaling Pathway Resulted in SUDEP

Adenosinergic activities are tightly linked to the etiological and pathophysiological outcomes of seizures and epilepsy (Ashraf et al., 2021; Faingold et al., 2016; Kommajosyula et al., 2016; Masino et al., 2014; O'Brien, 1988; Shen et al., 2014). Acute and chronic seizures can trigger repeated adenosine surges and increase adenosine tone in the brain, which can act as an endogenous anticonvulsant and also reset neuron network stability by affecting neurotransmission at the synapse (Masino et al., 2014; O'Brien, 1988). It is increasingly established that two main systems are contributing to the extracellular adenosine that engages the adenosine modulation system in the brain: the activity of equilibrative nucleoside transporters (ENTs) and of ADK mostly associated with a global A_1_R function and CD73-mediated formation of ATP-derived adenosine tightly associated with A_2A_R activation (Cunha, 2016). Seizure-induced adenosine surges can result in changes in ADK (Aronica et al., 2011), which together with adaptive alterations of the density and activity of adenosine receptors (He et al., 2020; Rebola et al., 2005c) maintain balanced adenosine activities in epileptic sites. Despite Adk^+/−^ mutants having reduced endogenous brain ADK, intriguingly, no overt genotype effect of Adk^+/−^ mutants was observed in IHKA-induced chronic seizures (Figure 1B). This could be attributed to potential adaptations of mutants to genetic knockdown of ADK during their development, or it could be masked by undetermined compensatory changes in the adenosinergic pathway: changes in additional adenosine metabolic pathways, e.g., adenosine deaminase (to process adenosine into inosine and then into hypoxanthine), and/or changes in adenosine receptors in the hippocampus of Adk^+/−^ mutants. These potential changes may separately and/or synergistically affect the seizure phenotypes in Adk^+/−^ mice. The complexity and seeming discrepancy of the relationship between the ADK/adenosine and seizure phenotypes were also shown in the Sandau et al. study with similar ADK deletion in the hippocampus (Sandau et al., 2016), in which the Adk^Δbrain^ mice (i.e., Nestin-Cre^+/−^:Adk^fl/fl^ mice) were used with the characterized conditional *Adk* gene deletion, which caused brain-wide ADK deficiency and increased synaptic adenosine levels. However, Adk^Δbrain^ mice surprisingly showed an increase in spontaneous seizures and susceptibility to seizure induction compared to their WT littermates (Sandau et al., 2016). The findings from Adk^Δbrain^ mice and our Adk^+/−^ mutants indicated a complexity between ADK/adenosine and seizure (onset) phenotypes, which is also a suggestive indication supporting the contention that studying only ADK as a controller of the adenosine levels may well be inadequate. The work of Sandau et al. (2016) did not consider the possibility that changes in ATP release and ect-nucleotidase activity might have occurred in the tested Adk transgenic mice. Nevertheless, whether genetic ADK knockdown can yield possible preconditioning effects against the development of epilepsy warrants additional evaluation.

In the brainstem, we demonstrated that chronic seizures led to a compensatory increase in NTS ADK (of WT mice), an area outside the hippocampal seizure origin; epileptic Adk^+/-^ mice did not experience any ADK level increases, which may have contributed to their increased death rates. The NTS level of A_2A_Rs was enhanced in epileptic mice along with increased cAMP, suggesting an increased output of A_2A_R activation. Notably, in epileptic Adk^+/-^ mice, the combination of impaired adenosine removal potential with increased A_2A_Rs in the NTS could result in lethal suppression on cardiorespiratory reflexes of chemo- and baroreceptors inputs (Wilson et al., 2004; Zoccal et al., 2014; Minic et al., 2015). Mechanistically, A_2A_Rs can alter GABAergic activities in several aspects: 1) A_2A_R controls the depolarization-evoked GABA release in synaptosomes in the hippocampus (Cunha and Ribeiro, 2000) and striatum (Kirk and Richardson, 1994); 2) A_2A_R controls the activity of GABAergic interneurons, increasing synchronization in hippocampal networks (Rombo et al., 2015) and most evidently controlling adaptive plastic changes in GABAergic synapses in the prefrontal cortex (Kerkhofs et al., 2018); 3) previous studies in different animal models of epilepsy suggested that the neuroprotection afforded by A_2A_R blockade might involve a rebalance of GABAergic transmission (Seo et al., 2020); 4) A_2A_R are critical mediators of the stability of GABAergic synapses (Gomez-Castro et al., 2021). Thus, overactivation of A_2A_Rs in the NTS can attenuate depolarization-evoked GABA release (Saransaari and Oja, 2005) and affect oscillations of GABAergic interneurons (Buzsáki and Wang, 2012). Meanwhile, it has been shown that A_2A_R is located in glutamatergic synapses (Rebola et al., 2005b) acting as a controller of plasticity processes at glutamatergic synapses, either in the hippocampus (Costenla et al., 2011), dorsal or ventral (Reis et al., 2019) hippocampus, the striatum (Shen et al., 2008; Li et al., 2015), the amygdala (Simões et al., 2016), or the prefrontal cortex (Kerkhofs et al., 2018). Overactivated A_2A_R may also lead to altered synaptic glutamatergic activities in NTS neurons.

Remarkably, A_2A_R antagonist SCH58261 disinhibited KA-induced continuous suppression in gamma oscillation and enhanced theta and beta oscillations in anesthesia preparation (Figure 5E), likely preventing the lethal suppression of NTS neuronal activity during and after seizures (Kuo et al., 2016). Theta, beta, and gamma oscillations not only represent activity changes of local neurons in the recorded area but also activities from specific brain circuits of information flow (Massimini et al., 2004). Our study demonstrated that under chronic epileptic condition, KA and/or acute seizure tends to decrease the slow-wave synchrony, suggesting decreased communications between brain regions. SCH58261 pretreatment can restore KA-induced inhibition on slow waves, indicating the disinhibition is mainly mediated by A_2A_R activation. In the mammalian cortex, neural communication is organized by 30–100 Hz gamma oscillation, with gamma frequency related to processing speed in neural networks (Insel et al., 2012). Furthermore, this fast band typically requires interplay between excitatory and inhibitory transmission (Buzsáki et al., 2012). However, we need to bear in mind that though LFP recorded from the anesthetized animal has been accepted in epilepsy studies (Williams et al., 2016; Wenzel et al., 2017) to reflect synchronous activities of neuronal assemblies, the anesthetized states might mask changes in seizure activity, respiration, and potential neurobehavioral outcomes. The LFP findings aimed to mechanistically demonstrate the local neuronal activities in the NTS, linked to IHKA-induced chronic epilepsy and/or affected by adenosine-A_2A_R activities.

In the NTS, neurons are segregated into neuronal clusters, which receive distinct cardiorespiratory afferents and regulate their function by regulating NMDAR- and GABAAR-mediated excitatory/inhibitory synaptic plasticity (Bantikyan et al., 2009). We propose that, in ADK^+/−^ mice, the inhibitory transmission may predominate in the NTS because of its impaired capacity to remove the endogenous adenosine, which may lead to a potential tonic suppression of cardiorespiratory function, and a higher vulnerability to SUDEP risk. As such, the additional KA injection can cause a higher sudden death rate in ADK^−/+^ mice. While the cell type- and/or neurotransmitter-dependent actions of A_2A_R in the NTS warrant further characterization to reveal the complexity of A_2A_R-mediated regulation in NTS circuits, a report from Derera *et al* (2017) supports the role of A_2A_R in the NTS in an increased risk of cardiorespiratory dysfunction and sudden death in TLE patients (Derera et al., 2017). Enhanced A_2A_R signaling may not be limited to NTS; it may affect other autonomic brainstem structures that potentially contribute to SUDEP. Our findings indicate a crucial role of A_2A_R in the pathophysiology of SUDEP and suggest that antagonism of A_2A_R may be a therapeutic strategy for SUDEP prevention.

### Adenosinergic Intervention—do the Timing and Subtype of Receptor Matter?

With a broader consideration, seizures also trigger a release of ATP (Wieraszko et al., 1989; Augusto et al., 2021) and an increased extracellular catabolism of ATP into adenosine (Bonan et al., 2000; Bruno et al., 2003; Nicolaidis et al., 2005), which sustains A_2A_R activation (Augusto et al., 2013; Carmo et al., 2019; Gonçalves et al., 2019; Alçada-Morais et al., 2021; Augusto et al., 2021) that is critically associated with seizure-induced neurodegeneration (Cognato et al., 2010; Canas et al., 2018; Augusto et al., 2021). This evidence is of key importance to understand the role of A_2A_R and the limitations in studying only the relation between ADK and adenosine neuromodulation without considering the whole limb of ATP release and ectonucleotidase activity selectively associated with A_2A_R activation (Augusto et al., 2013; Carmo et al., 2019; Gonçalves et al., 2019; Alçada-Morais et al., 2021; Augusto et al., 2021). Strangely, it has never been tested if decreased ADK results in aberrantly increased ATP release upon neuronal activation.

Further, due to the complexity of the adenosinergic actions in the CNS and diverse and wide distributions of A_1_R and A_2A_R across brain regions (O'Brien, 1988; Chen et al., 2014; Fredholm et al., 2005; Chen et al., 2013), the application of adenosinergic interventions to prevent SUDEP deserves deeper discussion. In the hippocampus, A_1_R suppresses the ictal firing of excitatory neurons; conversely, hippocampal A_2A_R activation is proconvulsant and proseizure (Zeraati et al., 2006; El Yacoubi et al., 2009). Clinical findings revealed upregulated hippocampal A_2A_Rs in patients with TLE, which supports the notion of applying A_2A_R antagonists without exacerbating epileptic seizures. In the brainstem, a working hypothesis is that A_2A_R activation leads to suppression of GABAergic inhibitory neurons and their mediated cardiorespiratory functions, whereas A_1_R activation promotes opposite effects (Phillis et al., 1997). These diverse effects of A_2A_R vs. A_1_R limit the potential application of the widely used, non-selective adenosine receptor antagonist, caffeine. Indeed, the cause-and-effect relationship between caffeine and epileptic seizures has long been debated (Kaufman and Sachdeo, 2003; Samsonsen et al., 2013) and was recently reviewed (Tescarollo et al., 2020) with due care to distinguish the effects of acutely administered caffeine compared to the ‘chronic’ consumption of caffeine, the latter attenuating epileptic-like phenotypes. Rather, specific antagonism of A_2A_R can reduce the adenosine surge-related brainstem suppression, while avoiding interference with A_1_R-mediated anticonvulsive actions. Last but not least, antiepileptic drug-resistant/refractory patients are linked to a high risk of SUDEP, while having tonic-clonic seizures is considered the greatest risk factor (Annegers and Coan, 1999). With the recent FDA approval of the A_2A_R antagonist istradefylline for PD treatment (Chen and Cunha, 2020), our results indicate istradefylline should be examined for repurposing for epileptic patients at risk of SUDEP. Together, A_2A_R antagonists may be a potential add-on anti-SUDEP approach. This approach may provide an important reduction of SUDEP with a remaining question as to the suppression of seizures.

## Data Availability

The raw data supporting the conclusions of this article will be made available by the authors, without undue reservation.
