# Supplementary material for: Adenosine-A2A Receptor Signaling Plays a Crucial Role in Sudden Unexpected Death in Epilepsy
Source: Front Pharmacol. 2022 Jun 9;13:910535. doi: 10.3389/fphar.2022.910535 (PMC9218562; doi:10.3389/fphar.2022.910535)
Supplement: Supplementary file 1 [file DataSheet1.DOCX]

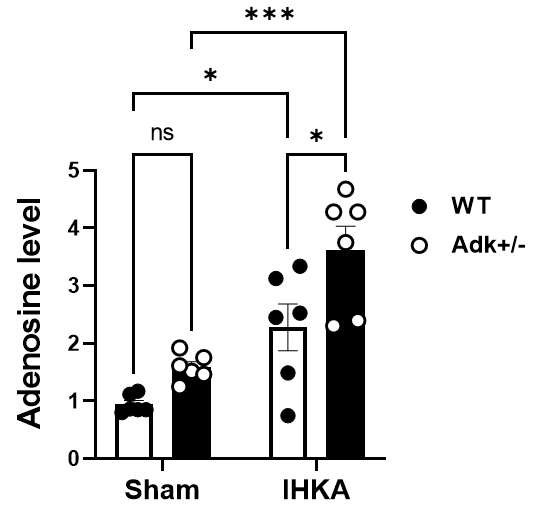


**Supplemental Figure 1. Adenosine levels in the brainstem of naïve and epileptic mice**

Adenosine (ADO) levels in the brainstem of IHKA modeled epileptic and Sham controls of both Adk +/- mutants were measured via HPLC along with corresponding WT controls (n = 6 / treatment/genotype). Samples were taken four weeks after IHKA-injection or intrahippocampal sham injection of saline (200 µL). The Two way ANOVA analysis showed a significant effect of treatment factor [F(1,20) = 32.17, p<0.0001] and a significant effect of genotype factor [F(1,20) = 11.17, p=0.0032]. Multiple comparisons of the HPLC data showed that the (i) Adk+/- mutants had a tendency of higher baseline brainstem adenosine level vs naïve WT mice, but not reached a statistical significance (p=0.4352, n= 6 per group); (ii) brainstem adenosine levels in epileptic Adk+/- mutants and WTs were both significantly higher vs their corresponding basal levels (p = 0.0005 and p = 0.0222, n = 6 per genotype per treatment); and (iii) importantly, the brainstem adenosine levels in epileptic Adk+/- mutants were significantly higher than epileptic WTs (p = p=0.0220, n= 6 per genotype). Data are mean ± SEM. *p<0.05, vs corresponding Adk+/- sham controls.

These data suggest that the Adk+/- mutant has reduced compensatory adaptation in adenosine removal under chronic epilepsy condition, which may contribute to the increased risk of SUDEP events in Adk+/- mutants.


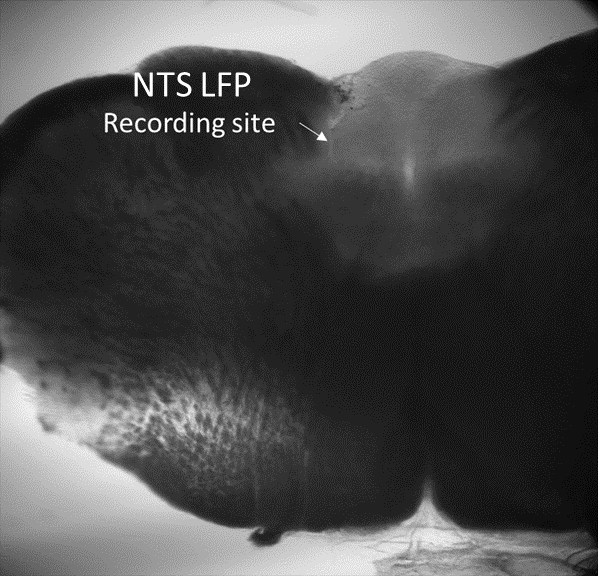


**Supplemental Figure 2. Demonstration of the NTS LFP recording site using unstained raw slides**.

Arrow indicating the track of electrode site for the NTS LFP recording.


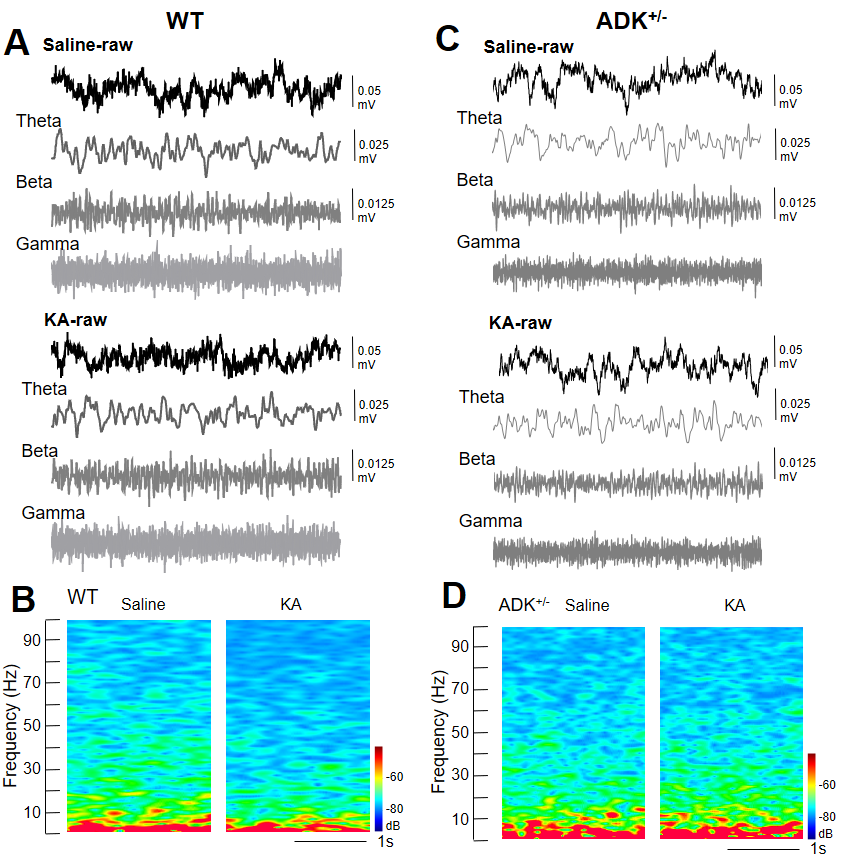


**Supplemental Figure 3. Raw trace and power spectrum for Adk^+/-^ and WT mice**

Typical LFP signals from one WT **(A)** or Adk^+/-^ **(B)** mouse after saline treatment (top) and subsequent KA injection (bottom). The first row shows a 5-sec of the raw trace (WT Saline-raw, upper panel; KA-raw, lower panel); the second to fourth rows show a 5-sec of filtered signals. The typical power spectrum of the above LFP signals was recorded after saline or KA injection in the WT **(C)** or Adk^+/-^ **(D)** mouse.
